# Supplementary material for: The impact of multiple abiotic stresses on ns-LTP2.8 gene transcript and ns-LTP2.8 protein accumulation in germinating barley (Hordeum vulgare L.) embryos
Source: PLoS One. 2024 Mar 19;19(3):e0299400. doi: 10.1371/journal.pone.0299400 (PMC10950244; doi:10.1371/journal.pone.0299400)
Supplement: S1 Fig — (DOCX) [file pone.0299400.s001.docx]

5’-GCCAGTACGTCAAGGACCCCAACTACGGGCACTACGTGAGCAGCCCACACGCG

CGCGACACCCTCAACTTGTGCGGCATACCCGTACCGCACTGCTAGCCGCCTAGC-3’

S1 Figure. *ns-LTP2.8* sequence amplified in RT-qPCR reaction, analysis done in triplicate
