# Supplementary material for: The impact of multiple abiotic stresses on ns-LTP2.8 gene transcript and ns-LTP2.8 protein accumulation in germinating barley (Hordeum vulgare L.) embryos
Source: PLoS One. 2024 Mar 19;19(3):e0299400. doi: 10.1371/journal.pone.0299400 (PMC10950244; doi:10.1371/journal.pone.0299400)
Supplement: S2 Fig — (DOCX) [file pone.0299400.s002.docx]

*EF1α UBI UPL GAPDH*

1.0

0.8

0.6

0.4

0.2

0.0


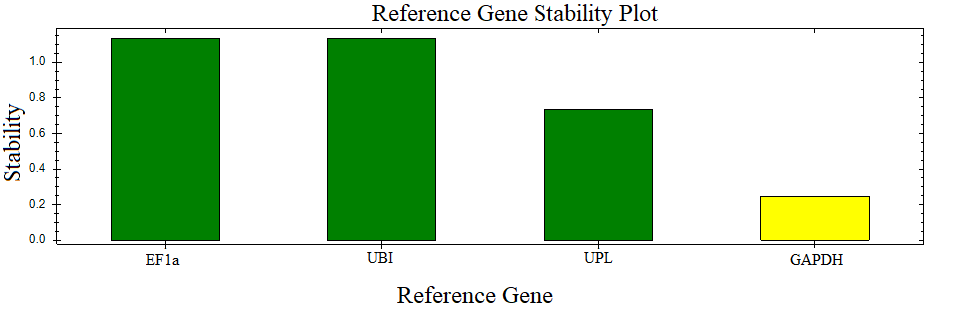


Stability

S2 Figure. Reference gene stability plot based on the built-in geNorm algorithm, calculated directly by the CFX Maestro software (Bio-Rad), analysis done in triplicate
