# Supplementary material for: The impact of multiple abiotic stresses on ns-LTP2.8 gene transcript and ns-LTP2.8 protein accumulation in germinating barley (Hordeum vulgare L.) embryos
Source: PLoS One. 2024 Mar 19;19(3):e0299400. doi: 10.1371/journal.pone.0299400 (PMC10950244; doi:10.1371/journal.pone.0299400)
Supplement: S3 Fig — Standard curves of ns-LPT2.8 (a) and reference genes (b, c) used for analysis of ns-LPT2.,8 mRNA content, calculated automatically by the CFX Maestro software (Bio-Rad). (DOCX) [file pone.0299400.s003.docx]

1. *ns-LTP2.8*


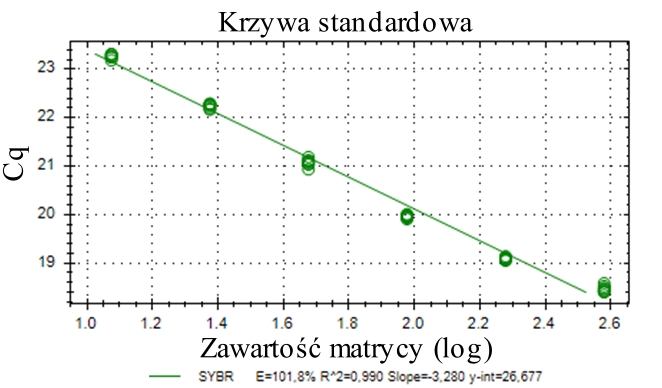


Cq

Template (log)

SYBR E=101,8% R^2=0,990 Slope=3,280

1. *EF1α*
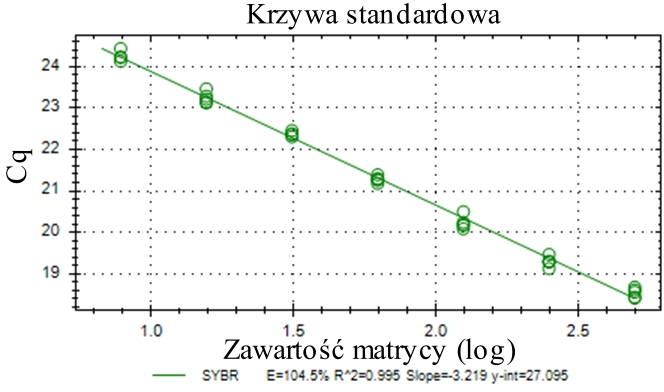


Cq

Template (log)

SYBR E=104,5% R^2=0,995 Slope=-3,219

1. *UBI*
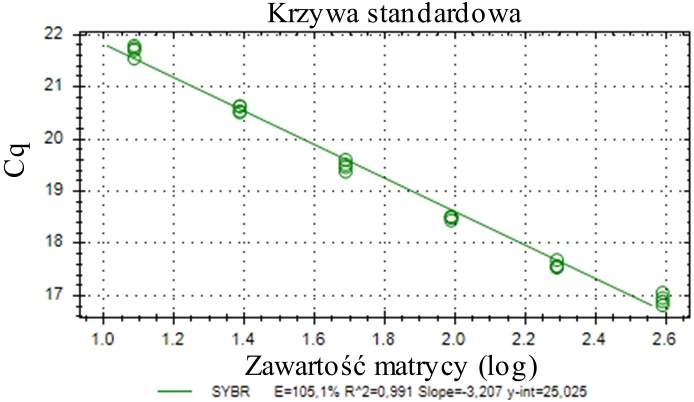


Cq

Template (log)

SYBR E=105,1% R^2=0,991 Slope=-3,207

S3 Figure. Standard curves of *ns-LPT2.8* (a) and reference genes (b, c) used for analysis of *ns-LPT2.,8* mRNA content, calculated automatically by the CFX Maestro software (Bio-Rad)
