# Supplementary material for: The impact of multiple abiotic stresses on ns-LTP2.8 gene transcript and ns-LTP2.8 protein accumulation in germinating barley (Hordeum vulgare L.) embryos
Source: PLoS One. 2024 Mar 19;19(3):e0299400. doi: 10.1371/journal.pone.0299400 (PMC10950244; doi:10.1371/journal.pone.0299400)
Supplement: S4 Fig — Error bars represent standard error of the mean, analysis done in triplicate. (DOCX) [file pone.0299400.s004.docx]

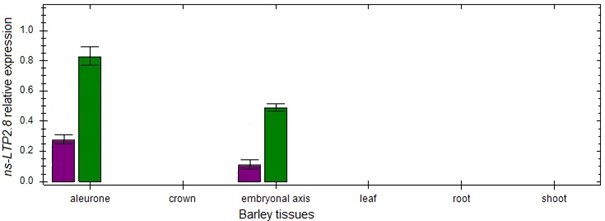

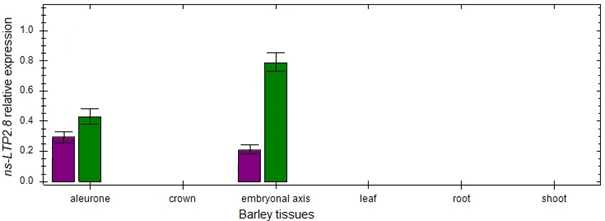


Maresi

CamB1

MCam53

MCam71


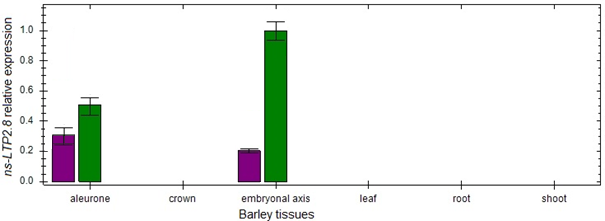

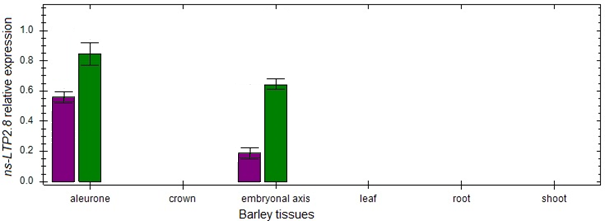


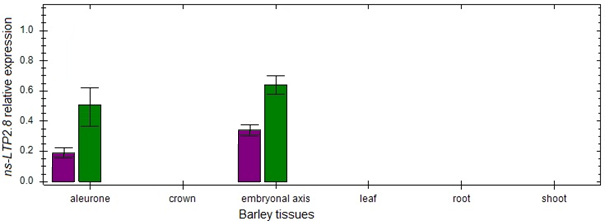

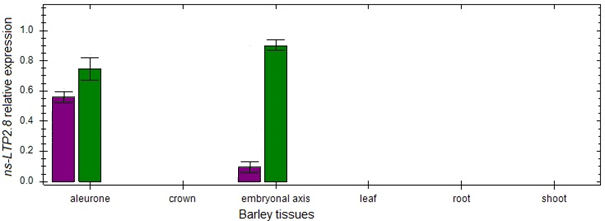


MCam87

MCam75


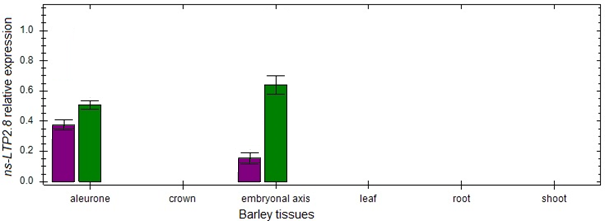

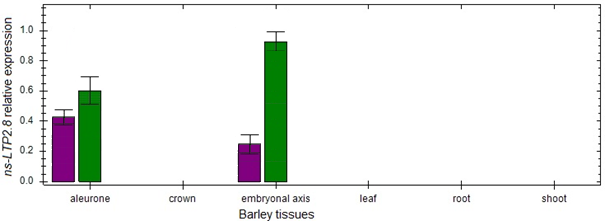


MPS37

MPS106


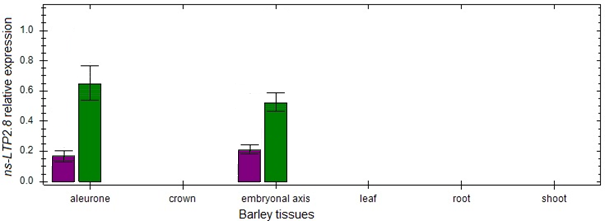

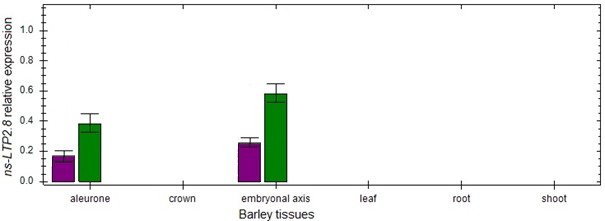


MPW14/9

MPW14/7


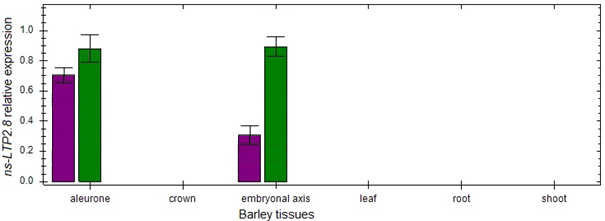

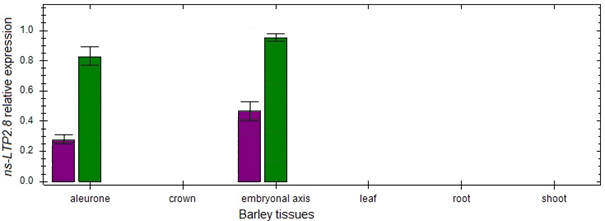


MPW15/4

MPW14/19

S4 Figure. Relative *ns-LTP2.8* expression in different barley tissues under optimal conditions (purple) and salt stress (green). Error bars represent standard error of the mean, analysis done in triplicate
