# Supplementary material for: The impact of multiple abiotic stresses on ns-LTP2.8 gene transcript and ns-LTP2.8 protein accumulation in germinating barley (Hordeum vulgare L.) embryos
Source: PLoS One. 2024 Mar 19;19(3):e0299400. doi: 10.1371/journal.pone.0299400 (PMC10950244; doi:10.1371/journal.pone.0299400)
Supplement: S5 Fig — Detection of ns-LTP2.8 (exemplary western blot signals) in the embryonal axis and aleurone (three biological replicates, top panel) vs. reference proteins ubiquitin, H3 histone (two replicates) and negative control–mature organs (bottom panel). (DOCX) [file pone.0299400.s005.docx]

ns-LTP2.8 ns-LTP2.8

(embryonal axis) .(aleurone)

1

1

3

2

3

2


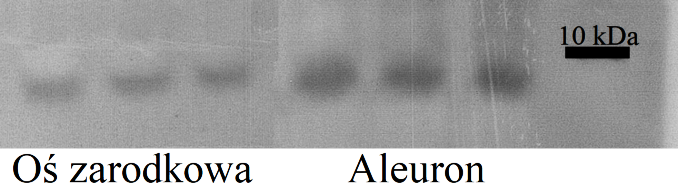


ns-LTP2.8

Ubiquitin Histone H3 (mature root (left)

(embryonal axis) (embryonal axis) leaf (right))

2

1

2

1

2

1


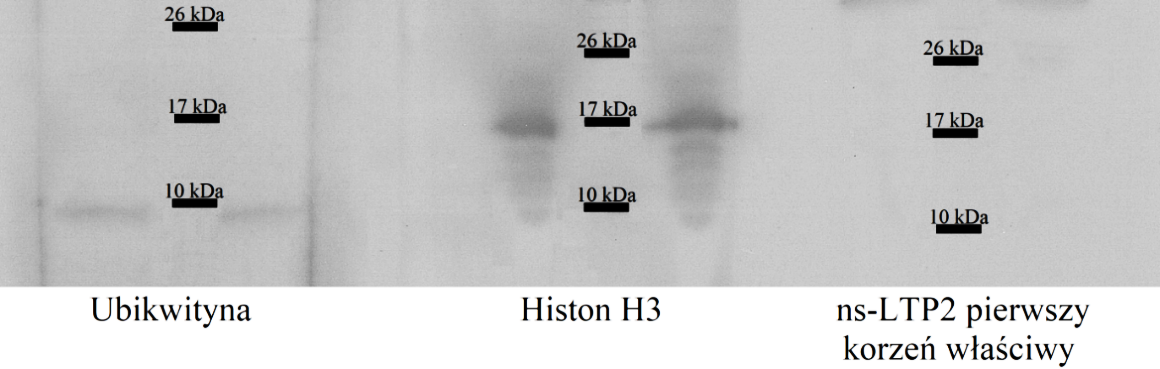


S5 Figure. Detection of ns-LTP2.8 (exemplary western blot signals) in the embryonal axis and aleurone (three biological replicates, top panel) vs. reference proteins ubiquitin, H3 histone (two replicates) and negative control – mature organs (bottom panel)
