# Supplementary material for: The impact of multiple abiotic stresses on ns-LTP2.8 gene transcript and ns-LTP2.8 protein accumulation in germinating barley (Hordeum vulgare L.) embryos
Source: PLoS One. 2024 Mar 19;19(3):e0299400. doi: 10.1371/journal.pone.0299400 (PMC10950244; doi:10.1371/journal.pone.0299400)
Supplement: S1 Table — a. Primers used for sequencing of ns-LTP2.8 coding sequence. b. Primers used for analysis of ns-LTP2.8 mRNA content. (DOCX) [file pone.0299400.s006.docx]

S1a Table. Primers used for sequencing of *ns-LTP2.8* coding sequence

| Gene | Sequence  5’→3’ | Ampl.  [bp] | T_m_ [°C] |
| --- | --- | --- | --- |
| *ns-LTP2.8* | F: GCGTGGCTGGCTACAAATAC  R: GTATGCCGCACAAGTTGAGG | ~410 | 58 |

S1b Table. Primers used for analysis of *ns-LTP2.8* mRNA content

| Gene | Sequence  5’→3’ | qRT-PCR  E [%] | R^2^ | Ampl.  [bp] | T_m_ [°C] | Reference |
| --- | --- | --- | --- | --- | --- | --- |
| reference genes | | | | | | |
| *EF1α* | F: CCTGGTATGGTTGTGACCTTTGG  R: GGGCTTGGTGGGAATCATCTTC | 104.5 | 0.995 | 408 | 63 | [1] |
| *UBI* | F: TCGCCGTCCTCCAGTTCTAC  R: CCTTCCTGAGCCTGGTTACCT | 105.1 | 0.991 | 63 | 63 | [2] |
| analysed gene | | | | | | |
| *ns-LTP2.8* | F: TGTGCCAGTACGTCAAGGAC  R: CTCGATCGGCTAGGCGGCTA | 101.8 | 0.990 | 118 | 62 | - |

[1] Al-Daoue A, Shoaib A, Al-Shehadah E, Jawhar M, Edin Arabi MI. Transcriptome analysis of the barley-*Rynhosporium secalis* interaction. Plant Pathol. J. 2014;30: 425-431.

[2] Rapacz M, Stępień A, Skorupa K. Internal standards for quantitative RT-PCR studies of gene expression under drought treatment in barley (*Hordeum vulgare* L.): the effects of developmental stage and leaf age. Acta Physiol. Plant. 2012;34: 1723.
